# Clowning in children undergoing potentially anxiety-provoking procedures: a systematic review and meta-analysis

**DOI:** 10.1186/s13643-019-1095-4

**Published:** 2019-07-19

**Authors:** Nadja Könsgen, Stephanie Polus, Tanja Rombey, Dawid Pieper

**Affiliations:** 1 Institute of Health Economics and Clinical Epidemiology, University of Cologne, Gleueler Straße 176-178, 50935 Cologne, Germany; 2 Institute for Research in Operative Medicine, Witten/Herdecke University, Ostmerheimer Str. 200, 51109 Cologne, Germany

**Keywords:** Clowning, Anxiety, Systematic review, Meta-analysis

## Abstract

**Background:**

The operation areas of clowns in the medical context are multifaceted. Clowning in children undergoing surgery has been shown to be able to lessen children’s anxiety. Hence, our aim was to assess the effectiveness of clowning on anxiety in children undergoing potentially anxiety-provoking procedures.

**Methods:**

We searched MEDLINE, CENTRAL, and EMBASE for randomized controlled trials (RCTs) in December 2018. The primary outcome was children’s anxiety. We used the Cochrane risk of bias tool to assess risk of bias of the included studies.

**Results:**

We found eleven RCTs including 733 children. Their risk of bias was relatively high.

Children undergoing clowning were significantly less anxious in preoperative time compared to parental presence or no intervention (mean difference (MD) − 7.16; 95% CI − 10.58, − 3.75) and in operation, induction, or patient room (MD − 20.45; 95% CI − 35.54, − 5.37), but not during mask application or physician examination (MD 2.33; 95% CI − 4.82, 9.48). Compared with midazolam, children’s anxiety was significantly lower in preoperative time (MD − 7.60; 95% CI − 11.73, − 3.47), but not in the induction room (MD − 9.63; 95% CI − 21.04, 1.77).

**Conclusions:**

Clowning seems to lower children’s anxiety, but because of the increased risk of bias of included studies and the very low quality of evidence, these results should be considered with caution.

**Systematic review registration:**

PROSPERO CRD42016039045

**Electronic supplementary material:**

The online version of this article (10.1186/s13643-019-1095-4) contains supplementary material, which is available to authorized users.

## Background

Distress and anxiety are common in children undergoing medical procedures. Fifty-three percent of children undergoing anesthesia are reported to suffer from high levels of anxiety [1]. Both, children and their parents, struggle with anxiety and distress even in case of minor surgery or routine procedures [2].

Potentially anxiety-provoking procedures such as anesthesia, immunization, catheterization, or dental treatments can cause negative physical and mental effects such as separation anxiety, sleeping, or eating disorders and are associated with a higher consumption of analgesics [3]. Anxiety was found to be the main factor in determining negative preoperative and postoperative effects [2]. Parental anxiety may serve as a predictor of children’s anxiety [4], as children accompanied by extremely anxious parents reflect their parents’ fears [5] or parents’ fears may reflect their child’s fear.

Preoperative anxiety in children can be reduced by pharmacological and behavioral interventions [6]. An effective behavioral intervention in decreasing patient pain can include the use of humor [7], such as clowning. Clowns are named differently, potentially reflecting different clown interventions: clown doctor, professional clown, medical clown, therapeutic clowns, and hospital clown. Clown doctors, for example, always work in pairs to free the child from the pressure to participate. Therapeutic clowns, on the other hand, work alone to mirror the child’s vulnerability [8].

A Cochrane review on non-pharmacological interventions for assisting the induction of anesthesia in children, including clowning, found a decrease in children’s anxiety in the operating/induction room when compared with parental presence, but not in comparison to midazolam [9]. However, the operation areas of medical clowns are multifaceted and there are several studies that examined the effect of clowning for different medical procedures: Goldberg et al. [10] found that clowning reduces children’s anxiety undergoing allergy skin prick test. Wolniez et al. [11] found that clowning seems to lower parental anxiety during intravenous access. Tener et al. [12] found that sexually abused children undergoing clowning during anogenital examination express less fear. Viggiano et al. [13] found that psychological interventions such as clowning during magnetic resonance imaging alleviate children’s anxiety and fear. Hansen et al. [14] found no effect of clowning in case of botulinum toxin injections in children treated for the first time. Meiri et al. [15] found that clowning lowers parental anxiety during venous blood drawing. These procedures have in common to be minor medical interventions and can potentially have the same effects on children’s anxiety. Therefore, the aim of this systematic review was to assess the effectiveness of clowning in children undergoing potentially anxiety-provoking procedures.

## Methods

This systematic review was registered in PROSPERO (CRD42016039045) and incorporated the Preferred Reporting Items for Systematic Reviews and Meta-Analyses (PRISMA) [16]. The PRISMA checklist including the page number for each item can be found in Additional file 1.

### Eligibility criteria

We considered studies including children aged 0 to 17 years undergoing potentially anxiety-provoking procedures. Any type of intervention including a physically present clown and any type of comparison were included. We included all studies stating any of the various names reflecting clown interventions and grouped them under the term “clowning” albeit named differently in the primary studies. We included as outcomes children’s anxiety, parental anxiety, children’s pain, negative postoperative behavior, and cooperation. Primary outcome was children’s anxiety defined and measured on the basis of criteria stated by the study authors. We included RCTs irrespective of their language. We excluded quasi-RCTs (qRCTs), because of the low number of qRCTs found in the Cochrane review [9] and their high risk of bias due to inadequate random sequence generation.

### Information sources

We performed a systematic literature search in December 2018 in the electronic databases MEDLINE (via PubMed), CENTRAL (via the Cochrane Library), and EMBASE (via EMBASE). Key terms included “children”, “pediatric”, “kids”, “infant”, “adolescent”, “clown”, “randomized controlled trial”, and “controlled clinical trial.” Further details of the search strategy are provided in Additional file 2. In addition, we performed reference checking of all included studies and we searched ClinicalTrials.gov for ongoing studies and for completed but not yet published studies in December 2018. We also contacted study authors for their study protocol.

### Study selection

Two reviewers (NK, DP, TR or SP) independently screened titles and abstracts and checked the full texts for inclusion. We resolved discrepancies in a discussion between the two reviewers or with the involvement of a third reviewer.

### Data collection process

We used a standardized form for data extraction. One reviewer (NK, TR) performed data extraction, and a second (DP, SP or TR) verified all extracted data. We resolved disagreements through discussion with the involvement of a third reviewer if necessary. We extracted data on author, year of publication, setting (i.e., inpatient or outpatient), inclusion criteria, type of potentially anxiety-provoking procedure, clown intervention, comparison, children’s age, and number of randomized cases. We extracted effect measures using mean value, standard deviation, and measuring instrument. We presented results as follows: mean difference, 95% confidence interval, and *I*^2^ if several studies were included in the meta-analysis. We considered *p* < 0.05 to be statistically significant.

Two studies only reported *p* values comparing intervention group and two comparison groups [17, 18]. Another study only reported *p* values < 0.01, without reporting exact *p* values [19]. We therefore recalculated *p* values for these studies based on the reported means, standard deviations, and number of assessed children.

One study did not report standard deviations regarding parental trait anxiety [10]. Therefore, standard deviations were imputed using averages of relevant candidate standard deviations [20]. We considered standard deviations of intervention groups and of comparison groups concerning parental trait anxiety relevant.

### Risk of bias in individual studies

The included studies were each independently evaluated by two reviewers (NK, DP, SP, TR) using the Cochrane risk of bias tool [20]. We evaluated the blinding of outcome assessment at the outcome level. We resolved discrepancies between the two reviewers; if necessary, we consulted a third. We considered the individual studies’ risk of bias in interpretation and conclusion.

### Synthesis of results

We used Review Manager (RevMan) 5.3 software to compute pooled effect estimates for children’s and parental anxiety. Principal summary measure was the difference in means with the corresponding 95% confidence interval. We used a random-effect model to compute mean differences (MDs) between clowning and the comparison groups. We measured heterogeneity for each meta-analysis using *I*^2^.

### Publication bias

For meta-analyses including 10 or more studies, we planned to assess publication bias creating a funnel plot.

### Additional analyses

We planned subgroup analysis regarding children’s age, setting, and type of clown intervention.

### Overall quality assessment

One reviewer (NK) assessed the overall quality of evidence using GRADEpro [21] for results from meta-analyses, and another one (TR) verified quality assessments.

## Results

### Deviation from protocol

We decided post-protocol to exclude studies examining physically absent clowns (e.g., on videos or apps) because we did not consider physically absent and physically present clowns to be comparable. We assumed that physically present clowns are able to interact more closely with the child and according to the various situations.

We also included two studies reporting only our secondary outcome parental anxiety [11, 22], one study that only reported the secondary outcome children’s pain [23], and another study reporting our secondary outcome cooperation [24].

We could not conduct subgroup analyses on children’s age, setting, and type of clown intervention due to the low number of studies included, but performed a posteriori a subgroup analysis of the different types of anxiety-provoking procedures.

Finally, we decided a posteriori to grade quality of evidence using GRADE.

### Study selection

**Fig. 1:**
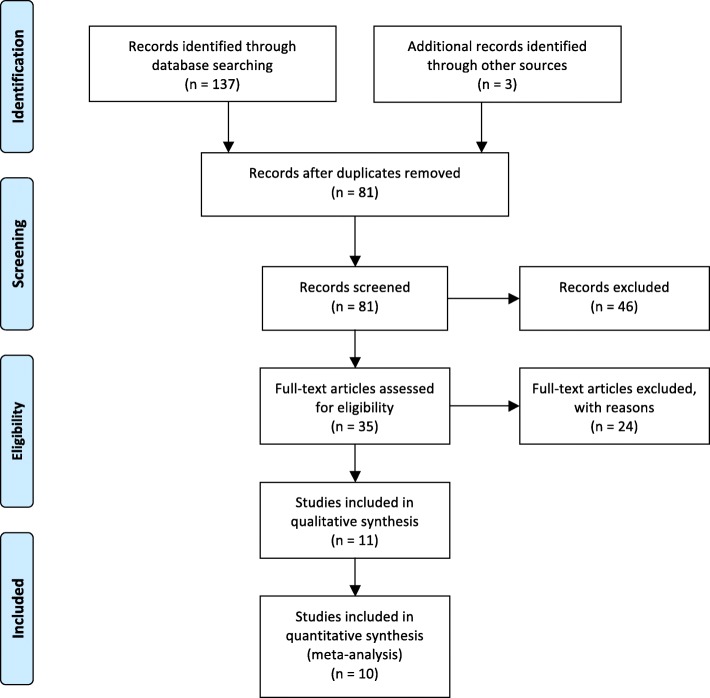
PRISMA flow diagram of the systematic literature search

Our searches in the electronic databases yielded a total of 137 studies; 3 additional studies were identified through reference checking [25–27]. Ultimately, 11 studies met our inclusion criteria. The selection process is illustrated in Fig. 1 (excluded studies are available in Additional file 3).

### Study characteristics

The studies included 733 children aged 2 to 17 years. Study size ranged from 40 to 120 randomized patients. The studies were conducted in Italy [4, 18, 22, 27], Israel [10, 11, 19, 23, 28], Turkey [24], and the USA [17]. Data were collected from June 2003 to September 2015. Seven studies were performed in an outpatient setting, one in an emergency department [23], another study in a hospital’s burn unit [24], and in two studies, the setting was unclear [11, 17]. Potentially anxiety-provoking procedure included anesthesia [4, 18, 19, 22, 27, 28], allergy skin prick test [10], physical examination [17], insertion of an intravenous catheter [11], burn dressing change [24], and blood tests or intravenous cannulation [23].

One of the included studies reported a conflict of interest for one of the involved authors [10]. Five studies declared no conflict of interest [4, 11, 19, 24, 28], and five studies did not comment on this [17, 18, 22, 23, 27]. However, seven studies received support by organizations, such as Anna Meyer Foundation that support hospital clowns, or by clown organizations [4, 10, 18, 19, 23, 27, 28]. Two studies reported receiving no financial support at all [11, 24]; the other two were supported by individuals or institutions not associated with clowning [17, 22].

Four of the eleven studies reported data on children’s anxiety only [17–19, 28], whereas two studies reported data on parental anxiety only [11, 22]. Three studies reported data on both outcomes [4, 10, 18], and only one study reported data on children’s pain [23]. Additionally, we found one RCT assessing children’s cooperation [24]. We did not find RCTs assessing negative postoperative behavior. We identified three different comparison groups: parental presence or no intervention, oral midazolam (a commonly administered sedative in preoperative time), and the child life program. The child life program aims to help children develop coping skills with two major components. One component is play which is intended to help children feel more comfortable; the other component is psychological preparation [29].

Differentiation between parental presence and no intervention was difficult because parents usually accompany their children. In two studies, parental presence was not mentioned [10, 17]. Due to the fact that young children were included, we assumed that they were accompanied by their parents and combined parental presence and no intervention to one comparison group. Three studies included two comparisons; the other eight studies included only one comparison. We included eleven comparisons between clowning and parental presence or no intervention [4, 10, 11, 17–19, 22–24, 27, 28]. Two studies compared clowning and oral midazolam [18, 19], and one clowning and the child life program [17]. All studies assessing children’s anxiety used the behavioral observation scale called Modified Yale Preoperative Anxiety Scale (m-YPAS) with a score from 0 to 100 and higher value meaning higher anxiety [30]. Parental trait and state anxiety was assessed based on self-report using the State-Trait Anxiety Inventory (STAI) with a score ranging from 20 to 80 on both trait and state anxiety and higher value meaning higher anxiety in all studies reporting this outcome [31]. Additionally, one study measured parental anxiety using a verbal rating scale with a score from 0 to 45 and higher value meaning higher anxiety [22]. Rimon et al. assessed children’s pain with the Faces Pain Scale–revised (FPS-R) for children aged four to seven [32] and a visual analog scale (VAS) for children over the age of seven and combined all scores to an overall mean pain score. Yildirim et al. [24] measured cooperation using a questionnaire and a child observation form on a scale from 0 to 16 with 16 meaning worst cooperation. Detailed information on the study characteristics and outcomes are depicted in Additional files 4 and 5.

Five studies had previous performance of a comparable procedure as an exclusion criterion [4, 10, 18, 19, 28]. Three studies reported the number of patients being previously treated with a painful procedure in the comparison groups [11, 23, 24]. Three studies did not report on previous comparable procedures [17, 22, 27].

### Risk of bias within studies

**Fig. 2:**
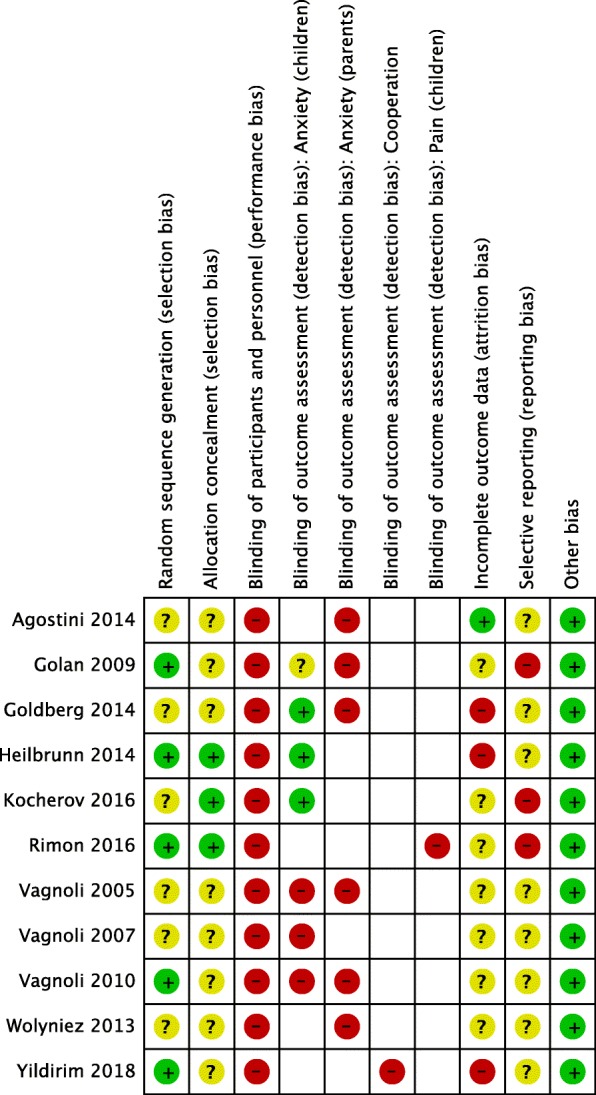
Risk of bias summary. “+”, low risk of bias; “?”,unclear risk of bias; “-”, high risk of bias. Uncoded boxes indicate that these studies did not include the corresponding outcome

For risk of bias assessment of individual studies see Fig. 2. Six studies had an unclear risk of selection bias regarding the sequence generation, as their methods of sequence generation were not described in sufficient detail; it was low in the remaining five studies. Risk of selection bias regarding allocation concealment was low in three studies and unclear in eight studies. Blinding of participants and personnel was not possible, and we therefore classified all studies as having high risk of bias. The risk of detection bias was graded to be high in three studies, unclear in one, and low in three studies regarding outcome assessment of children’s anxiety. Studies with a low risk of detection bias had filmed the children and after that evaluated videos without any evidence of the clown’s presence. We graded all studies assessing parental anxiety with a high risk of detection bias, because the STAI instrument and a verbal rating scale are self-reporting instruments and clowning was visible to parents. Detection bias was also high regarding children’s pain, as self-reporting scales were used for outcome assessment. Regarding children’s cooperation, detection bias was high as blinding was not mentioned and it would have been hardly possible. We graded risk of attrition bias high in three, low in one, and unclear in seven studies. We contacted all study authors and asked to send a study protocol, but only received a protocol for one study [23]. The risk of selective reporting was judged to be at least unclear for all studies missing a protocol, but three studies even had a high risk of reporting bias. We found no other sources of bias in any study; thus, the risk of other bias was low in all the included studies.

### Data analysis

We included ten studies in our meta-analysis. Studies synthesized in the meta-analysis used the same scales for measuring their outcomes, although this was not a prerequisite. Thus, we chose mean difference as effect estimate in all comparisons as all studies relied on the same scales. Six studies provided data concerning anxiety in children, five studies concerning parental anxiety, one concerning pain in children, and another one concerning children’s cooperation. We had to exclude one study from meta-analysis and report the available results narratively, as it reported outcome data using figures and *p* values without giving means and standard deviations [28].

### Children’s anxiety

#### Clowning vs. parental presence or no intervention (Fig. 3)

During preoperative time, pooled estimated effects were significantly in favor of clowning (MD = − 7.16 [− 10.58, − 3.75], *I*^2^ = 0%). In the operation, induction, or patient room, clowning was also significantly more effective than parental presence or no intervention (MD = − 20.45 [− 35.54, − 5.37], *I*^2^ = 93%). During mask application or physical examination, however, parental presence or no intervention seemed to be more effective in reducing children’s anxiety (MD = 2.33 [− 4.82, 9.48], *I*^2^ = 52%). During the process from waiting room until skin prick test, clowning was significantly more effective than parental presence or no intervention in one study (MD = − 13.80 [− 21.28, − 6.32]). We found similar results in Kocherov et al. where children undergoing clowning demonstrated significantly lower preoperative (*p* = 0.032) and postoperative anxiety (*p* = 0.004) than children receiving only parental presence or no intervention.

**Fig. 3:**
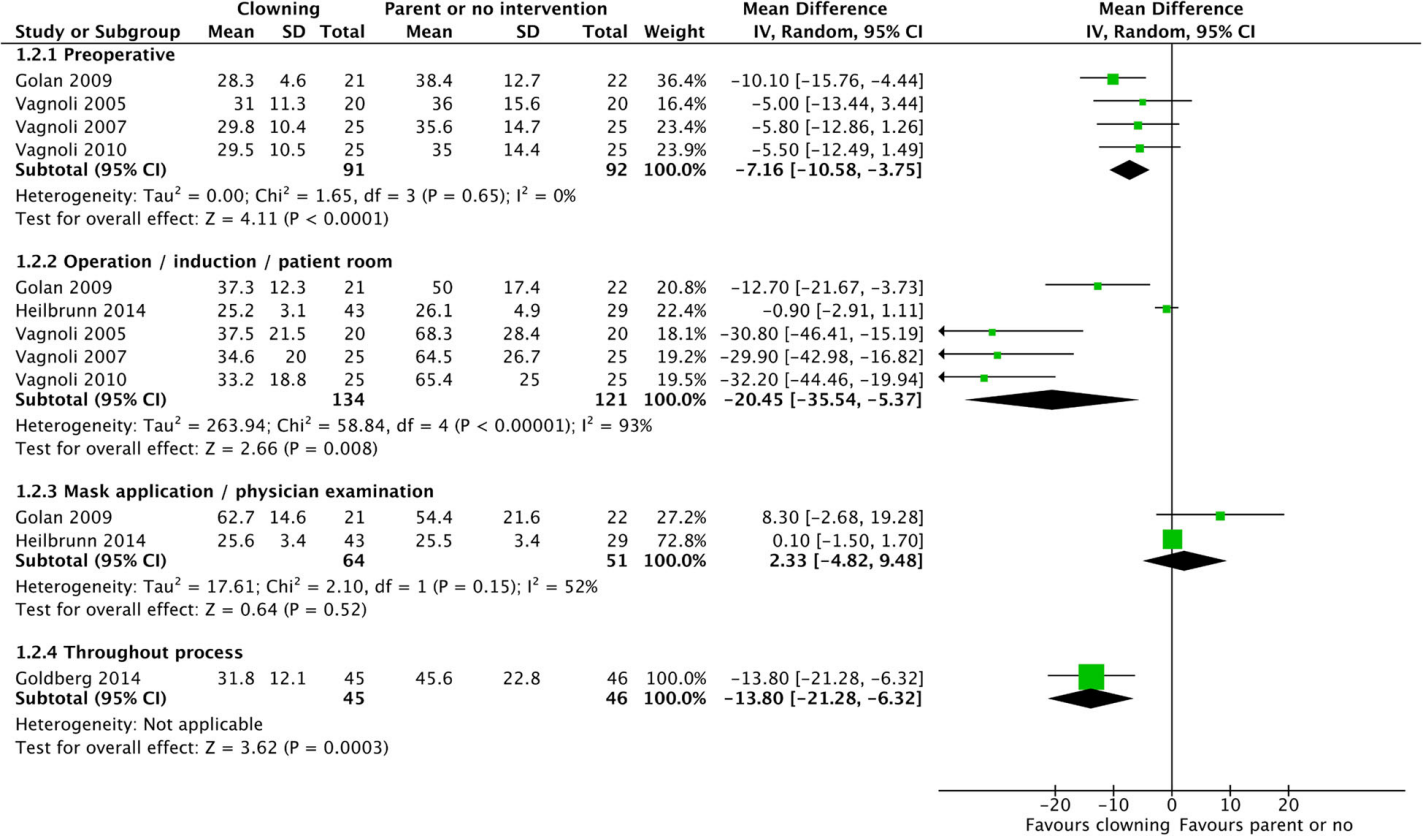
Children’s anxiety—clowning vs. parental presence or no intervention

We performed a sensitivity analysis to assess the impact of the inclusion of the two studies not explicitly mentioning parental presence. When excluding the two studies, clowning was even more effective in reducing children’s anxiety in operation, induction, or patient room (MD = − 25.55 [− 36.27, − 14.83], *I*^2^ = 68%).

#### Clowning vs. midazolam (Fig. 4)

In the preoperative period, pooled estimated effects were significantly in favor of clowning compared to midazolam (MD = − 7.60 [− 11.73, − 3.47], *I*^2^ = 0%). In the induction room, pooled estimated effects were in favor of clowning, but not statistically significantly (MD = − 9.63 [− 21.04, 1.77], *I*^2^ = 66%). During mask application, midazolam was significantly more effective in reducing children’s anxiety than clowning (MD = 12.80 [3.65, 21.95]) in one study.

**Fig. 4:**
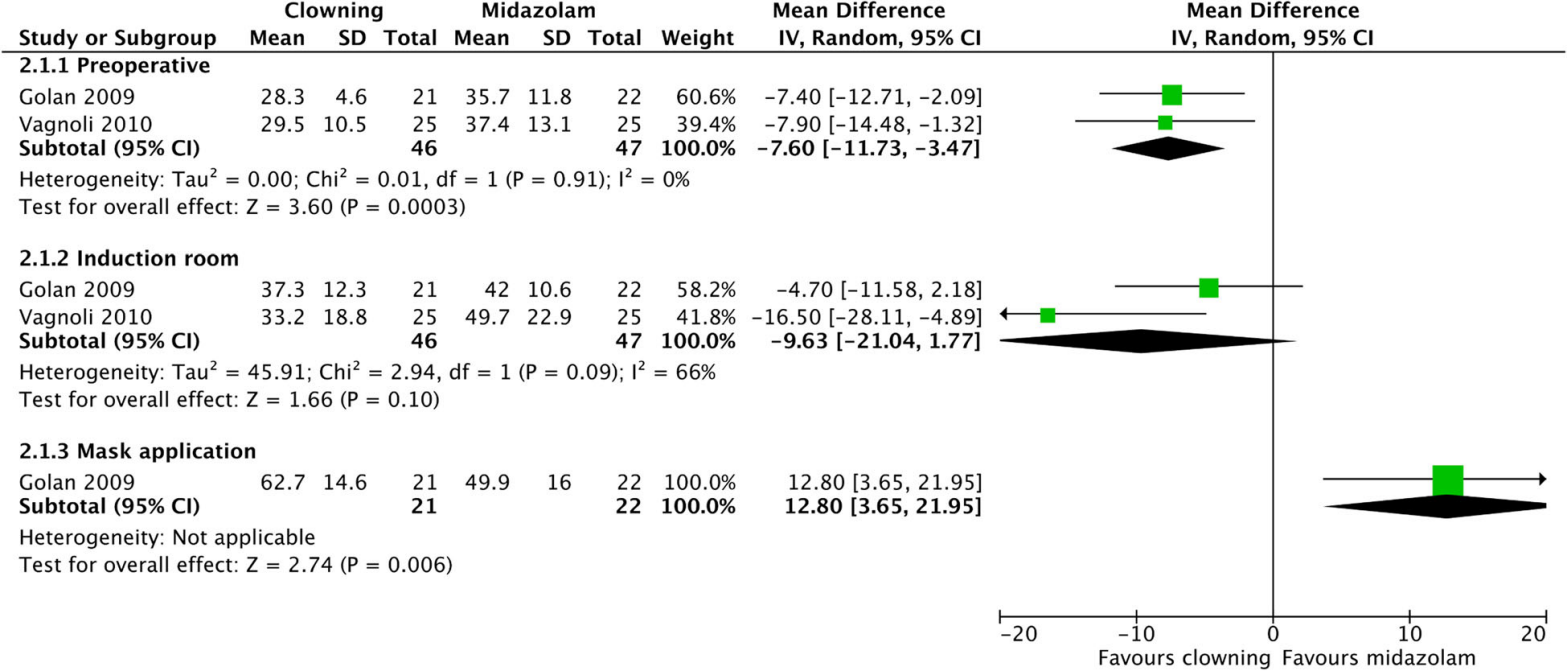
Children’s anxiety—clowning vs. midazolam

#### Clowning vs. child life program (Fig. 5)

One study compared clowning and the child life program. In the patient room, the child life program was significantly more effective than clowning (MD = 1.40 [0.25, 2.55]). During physical examination, the child life program was more effective, but not statistically significantly (MD = 1.20 [− 0.11, 2.51]).

**Fig. 5:**
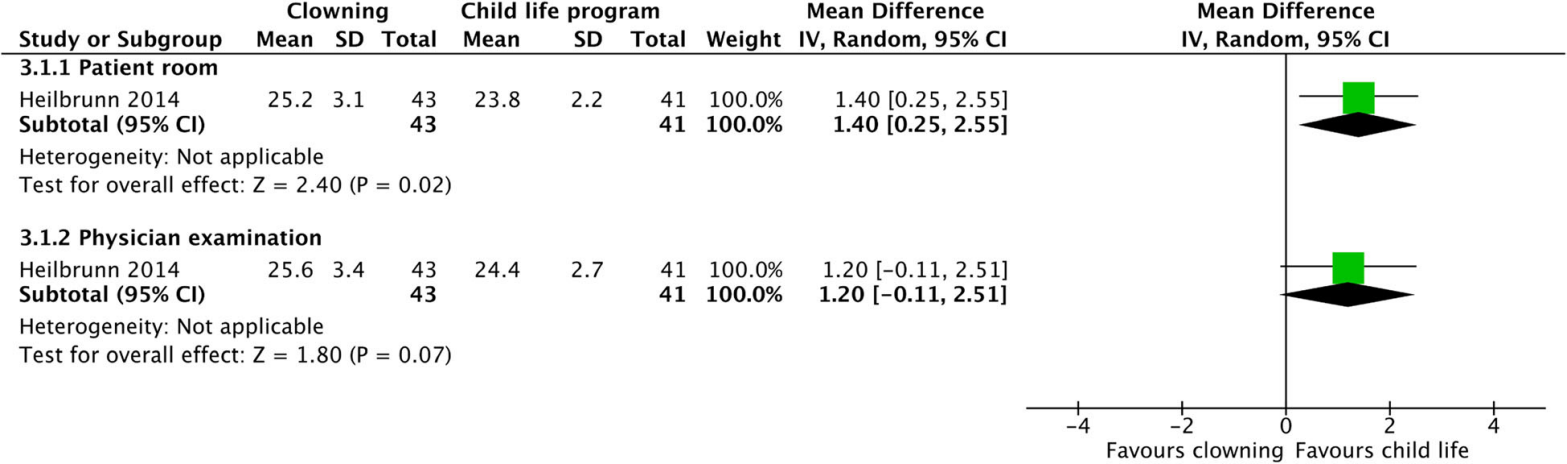
Children’s anxiety—clowning vs. child life program

### Parental anxiety

#### Clowning vs. parental presence or no intervention (Fig. 6)

Pooled estimated effects were significantly in favor of clowning compared to parental presence or no intervention for parental state anxiety (MD = − 4.00 [− 6.35, − 1.65], *I*^2^ = 0%) and for parental trait anxiety (MD = − 3.67 [− 6.65, − 0.69], *I*^2^ = 0%). Furthermore, clowning significantly decreased parental anxiety when measured on a verbal rating scale (MD = − 1.40 [− 2.40, − 0.40]). After excluding two studies which did not explicitly mention parental presence, clowning did not statistically significantly lower parental state anxiety (MD = − 2.83 [− 6.61, 0.36], *I*^2^ = 0%). However, clowning was still significantly decreasing parental trait anxiety (MD = − 4.45 [− 7.95, − 0.95], *I*^2^ = 0%).

**Fig. 6:**
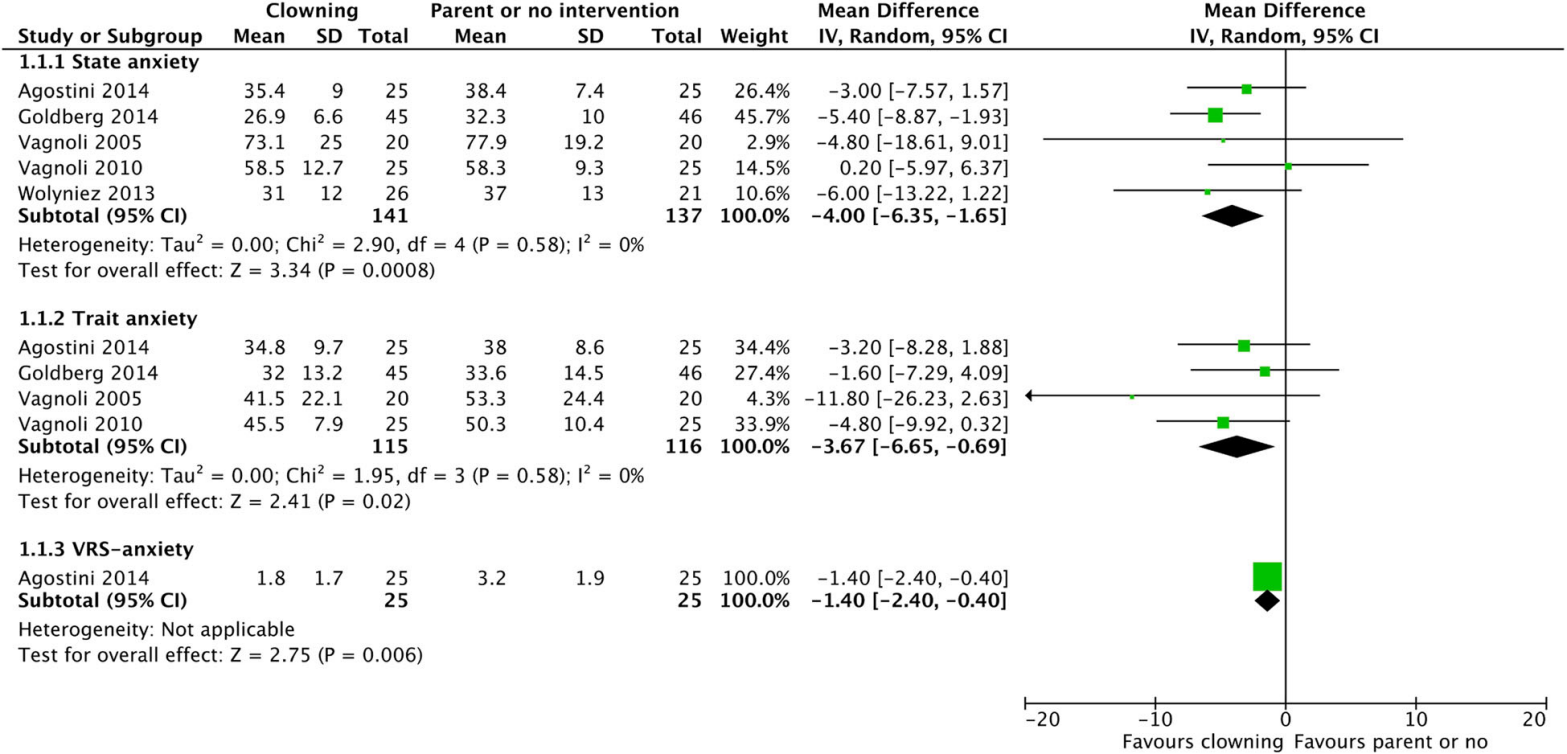
Parental anxiety—clowning vs. parental presence or no intervention

#### Clowning vs. midazolam (Fig. 7)

One study compared parental anxiety in children undergoing clowning and in children taking midazolam. Midazolam was significantly more successful in decreasing parental state anxiety than clowning (MD = 21.10 [13.95, 28.25]). Clowning seemed to be more effective in decreasing parental trait anxiety, but not statistically significantly (MD = − 4.20 [− 13.70, 5.30]).

**Fig. 7:**
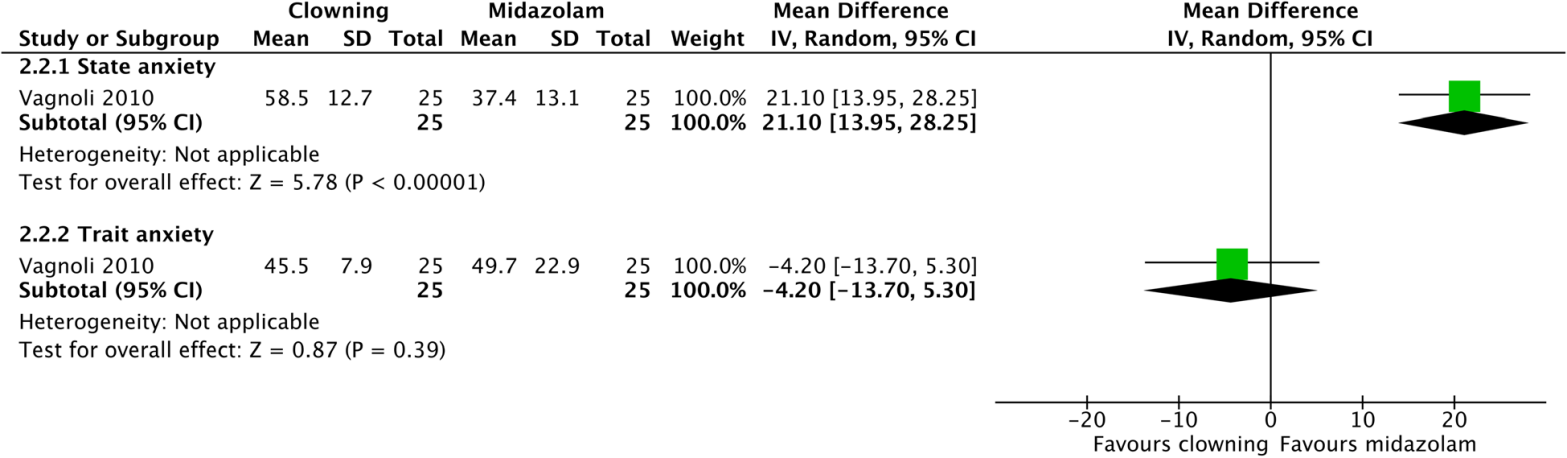
Parental anxiety—clowning vs. midazolam

#### Children’s pain (Fig. 8)

One study compared pain in children undergoing clowning and in children accompanied by at least one parent. Clowning was significantly more successful in decreasing children’s pain, which was measured 1 min after the procedure (MD = − 5.30 [− 6.77, − 3.83]).

**Fig. 8:**
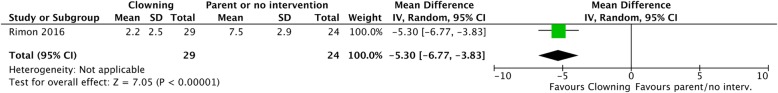
Children’s pain—clowning vs. parental presence or no intervention

#### Children’s cooperation (Fig. 9)

One study compared the behavior of children undergoing clowning and children accompanied by their parent. Children undergoing clowning had significant better cooperation than children with parental presence or no intervention (MD = − 6.20 [− 8.64, − 3.76]) [24].

**Fig. 9:**
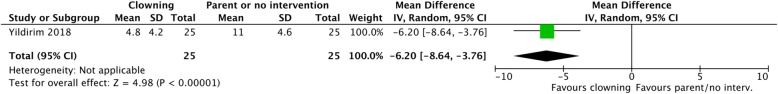
Children's cooperation-clowning vs. parental presence or no intervention

### Publication bias

Since there were less than ten studies per meta-analysis, assessment of publication bias using a funnel plot was not reasonable. We found four of the included studies registered on ClinicalTrials.gov [10, 11, 19, 23], and a protocol was available for one study [23]. We identified eight ongoing RCTs on clinicaltrials.gov (IDs: NCT02199587, NCT00886314, NCT01622218, NCT02701322, NCT02668679, NCT03122015, NCT03324828, NCT03671317) (see Additional file 6). One study started in 2018; therefore, we did not request this study’s results. Four authors did not respond to our request for further information. These studies had started in 2009, 2014, 2016, and 2017. One study was completed in 2012, and the study author replied that clowning seems to have no impact. Another study started in 2016 and was ceased due to technical issues. Last but not least, one study that started in 2017 did not provide sufficient contact information. Publication bias can therefore not be ruled out.

### Additional analysis

We assessed the different types of potentially anxiety-provoking procedures. Regarding anesthesia, clowning was able to reduce children’s anxiety significantly at least at one point of time [4, 18, 19, 27, 28], but did not report a statistically significant decrease of parents’ anxiety [4, 18, 22]. During mask application, children’s anxiety was significantly higher in the clowning group compared to midazolam [19]. One study that examined the effect of clowning in children undergoing allergy skin prick test found a statistically significant decreasing influence on children’s anxiety and parental state anxiety, but not for parental trait anxiety [10]. One study compared clowning with the child life program and parental presence or no intervention in children undergoing physical examination [17]. At both times, in the patient room and during examination, anxiety was higher in the clown group compared to the child life program, but only in the patient room statistical significance was reached. In comparison to parental presence or no intervention, anxiety was not significantly lower in patient room and not significantly higher during examination. Regarding intravenous access, one study showed a non-statistically significant reduction of parental state anxiety [11], while another study found that children’s pain scores were significantly lower in the medical clown group (*p* < 0.001) [23]. For burn dressing change, children undergoing clowning were significantly more cooperative than children with parental presence or no intervention [24].

### Overall quality of evidence

GRADE assessments yielded very low quality of evidence for all outcomes. For a summary of main findings comparing clowning and parental presence or no intervention, see Table 1. For a summary of main findings regarding other comparisons, see Additional files 7 and 8.

**Table 1:** Summary of main findings comparing clowning and parental presence or no intervention

Clowning compared to parental presence or no intervention in children undergoing potentially anxiety-provoking procedures						
Patient or population: children undergoing potentially anxiety-provoking procedures Setting: any setting Intervention: clowning Comparison: parental presence or no intervention						
Outcomes	Anticipated absolute effects^*^ (95% CI)		Relative effect (95% CI)	№ of participants (studies)	Certainty of the evidence (GRADE)	Comments
	Risk with parental presence or no intervention	Risk with clowning				
Children’s anxiety during preoperative time Assessed with m-YPAS Scale: from 0 to 100	–	The mean children’s anxiety during preoperative time in the intervention group was 7.16 lower (10.58 lower to 3.75 lower)	–	183 (4 RCTs)	⨁◯◯◯ Very low^a,b,c,d,e,f^	–
Children’s anxiety in operation/induction/patient room Assessed with mYPAS Scale: from 0 to 100	–	The mean children’s anxiety in operation/induction/patient room in the intervention group was 20.45 lower (35.54 lower to 5.37 lower)	–	255 (5 RCTs)	⨁◯◯◯ Very low^a,b,c,d,e,f,g^	–
Children’s anxiety during mask application/physician examination Assessed with m-YPAS Scale: from 0 to 100	–	The mean children’s anxiety during mask application/physician examination in the intervention group was 2.33 higher (4.82 lower to 9.48 higher)	–	115 (2 RCTs)	⨁◯◯◯ Very low^a,f,h,i,j^	–
Children’s anxiety throughout process Assessed with m-YPAS Scale: from 0 to 100	–	The mean children’s anxiety throughout process in the intervention group was 13.8 lower (21.28 lower to 6.32 lower)	–	91 (1 RCT)	⨁◯◯◯ Very low^a,c,d,f,i^	–
Parental anxiety-state anxiety Assessed with STAI Y-1 Scale: from 20 to 80	–	The mean parental anxiety-state anxiety in the intervention group was 4 lower (6.35 lower to 1.65 lower)	–	278 (5 RCTs)	⨁◯◯◯ Very low^a,b,c,d,e,f^	–
Parental anxiety-trait anxiety Assessed with STAI Y-2 Scale: from 20 to 80	–	The mean parental anxiety-trait anxiety in the intervention group was 3.67 lower (6.65 lower to 0.69 lower)	–	231 (4 RCTs)	⨁◯◯◯ Very low^a,b,c,d,e,f^	–
Parental anxiety Assessed with VRS scale Scale: from 0 to 45	–	The mean parental anxiety in the intervention group was 1.4 lower (2.4 lower to 0.4 lower)	–	50 (1 RCT)	⨁◯◯◯ Very low^a,c,d,e,f^	–
Children’s pain Assessed with combined score of FPS-R and VAS	–	The mean children’s pain in the intervention group was 5.3 lower (6.77 lower to 3.83 lower)	–	53 (1 RCT)	⨁◯◯◯ Very low^a,b,e,f,j^	–
Children’s cooperation Assessed with questionnaire and child observation form Scale: from 0 to 16	–	The mean children’s cooperation in the intervention group was 6.2 lower (8.64 lower to 3.76 lower)	–	50 (1 RCT)	⨁◯◯◯ Very low^a,c,d,e,f,i^	–

*The risk in the intervention group (and its 95% confidence interval) is based on the assumed risk in the comparison group and the relative effect of the intervention (and its 95% CI)

*CI* confidence interval, *MD* mean difference

GRADE Working Group grades of evidence

High certainty: we are very confident that the true effect lies close to that of the estimate of the effect.

Moderate certainty: we are moderately confident in the effect estimate. The true effect is likely to be close to the estimate of the effect, but there is a possibility that it is substantially different.

Low certainty: our confidence in the effect estimate is limited. The true effect may be substantially different from the estimate of the effect.

Very low certainty: we have very little confidence in the effect estimate. The true effect is likely to be substantially different from the estimate of effect.

^a^High risk of performance bias across the studies reporting this outcome

^b^Unclear risk of attrition bias across the studies reporting this outcome

^c^Unclear risk of reporting bias across the studies reporting this outcome

^d^Unclear risk of selection bias across the studies reporting this outcome

^e^High risk of detection bias across the studies reporting this outcome

^f^Sample size less than 400

^g^Considerable heterogeneity

^h^Unclear risk of detection bias across the studies reporting this outcome

^i^High risk of attrition bias across the studies reporting this outcome

^j^High risk of reporting bias across the studies reporting this outcome

## Discussion

### Summary of main findings

Clowning was effective to reduce children’s preoperative anxiety when compared to parental presence or no intervention. Results seem similarly to be in favor of clowning when compared with Midazolam; however, this should be taken with caution due to the small amount of studies assessed. Clowning was also shown to be effective in reducing parental anxiety, although the effect was rather small. Interestingly, clowning both lowered parental state and trait anxiety. Broadly speaking, parental state anxiety is a short-term measure of anxiety referring to a current reaction and trait anxiety is a long-term measure of anxiety based on personal characteristics [33]. Since the time of meeting between clowns, children, and their parent was rather short, it is interesting that clowning was able to lower parental trait anxiety as a stable trait. Intuitively, no influence of clowning on parental trait anxiety was expected. Of course, the effect could be a result of chance.

Owing to the moderate to high risk of bias of included studies and the very low quality of evidence, results should be taken as tentative. Moreover, the three studies by Vagnoli et al. were very homogeneous using identical inclusion criteria, the same potentially anxiety-provoking procedure, and the same setting. Analyzed outcomes were similar and were measured at the same time with the same instruments [4, 18, 27].

As the other identified RCTs were quite heterogeneous and assessed outcomes at different points of time, we also had to report results based on single studies. The included studies used different names for their clown intervention. However, it was unclear if the names were used appropriately. Therefore, we conducted no detailed analysis between the different types of clown interventions. It would be interesting to see if there are differences in effectiveness between the different types of clown interventions, though. Furthermore, only five studies listed previous comparable procedures as an exclusion criterion. Previous treatment with a comparable procedure can influence the effect of intervention.

### Limitations

This review has several limitations. Inevitably for systematic reviews, the results of this review are limited by the methods of the included studies. We only included RCTs and excluded qRCTs in this review for the increased risk of selection bias based on selective allocation [34]. It is possible that we included studies that were not recognizable as qRCTs due to insufficient reporting while we may have excluded studies with higher reporting quality, which acknowledged that their allocation method was not truly random. As one reviewer extracted data while another verified the extracted data, there might be a higher risk of inaccuracy compared to independent data extraction, although to the best of our knowledge, there is no empirical evidence that supports this assumption.

### Comparison with other systematic reviews

A high-quality Cochrane review assessing non-pharmacological interventions in children during induction of anesthesia reported that clowning, compared to parental presence, significantly reduced children’s anxiety in the induction room [9]. Our findings confirm the results of Manyande et al., despite the different study pool. Differences in the study pool are due to the fact that we also included studies with other interventions than anesthesia [10, 11, 17, 23, 24] and such that reported data on parental anxiety only [22]. We included two additional studies meeting the Cochrane review’s inclusion criteria [27, 28], while the Cochrane review included two additional studies that were qRCTs [35, 36]. These studies contained additional information about negative postoperative behavior that was not dealt with in any of our included studies. One reported a significant decrease in negative postoperative behavior in comparison to parental presence [35], and the other a significant higher affectivity, but a lower arousal in the clown group compared to parental presence [36]. These results were confirmed by a recently published updated systematic review [37].

Another systematic review showed several methodical differences compared to our review [38]. Most importantly, the authors did not differentiate between the different time points used in the included studies without giving a rationale. Our review revealed different effects for the different time points, though.

### Implications for research

High-quality RCTs are needed to draw conclusions about the effectiveness of clowning on children’s anxiety during medical procedures. Although blinding of outcome assessment was difficult, some studies filmed the children and edited the videotapes so that the clown’s presence was concealed when children’s anxiety was evaluated. However, blinding of participants and personnel was not possible due to the intervention’s nature. In general, study protocols should be published in advance to minimize the risk of selective reporting. It would be interesting to see if the results of independently-funded studies were different. We identified ongoing studies that could give further insights whether clowning is effective in children undergoing endocrine tests, surgery, and videofluoroscopic examination.

In addition, the evaluation of further outcomes would be desirable. Most of the included studies reported children’s or parental anxiety. But none of the RCTs reported negative postoperative behavior, and only one addressed children’s pain or cooperation. Uncontrolled pain may cause long-term negative emotional consequences [39], though, and in the induction of anesthesia, previously cooperative children may protest or try to struggle [40]. This can lead to an increase of anesthesia induction time and to traumatic experiences. In 47% of children undergoing surgery, problematic behavioral changes were seen at any of the observation times [41].

We found one study that reported a correlation between intervention effectiveness and children’s age. Clowning was more effective in reducing parental anxiety if the children were 8 years or older [11]. However, the question up to which age children can benefit from clowning is more important in this context. Our included studies had broad age ranges from 0 to 17 years.

Implementation of clowning in clinical practice is an important issue. On the one hand, clowning seems to be effective, but on the other hand, the clown’s acceptance by medical staff might be limited. One study assessing medical staff opinions about clowning found that the majority felt disturbed by the clown [4]. Our literature search identified two studies that assessed the opinion of a clown video or a clown app [42, 43]. A clown video or clown app could save money and time and prevent medical staff from feeling disturbed by the clown’s presence. More studies would be helpful to assess the effectiveness of clown technology particularly compared to physically present clowns.

### Implications for practice

Integration of clowning in clinical routine seems to be challenging because clowning leads to increased costs and resources compared to parental presence solely. There is a difference between a professional clown and a doctor masquerading as a clown regarding the height of costs, the type of staff that is additionally needed, and probably the effectiveness. Clown videos or clown apps could be a resource-effective alternative if they are shown to be effective. Cost-effectiveness analyses should be conducted in the future.

## Conclusion

Clowning in children undergoing potentially anxiety-provoking procedures seems to decrease not only children’s but also parents’ anxiety. Due to the moderate to poor quality of the included studies, RCTs of higher methodological quality would be desirable.

## Additional files

Additional file 1: PRISMA checklist. (DOC 32 kb)

Additional file 2: Search strategy. (DOCX 20 kb)

Additional file 3: Excluded studies. (DOCX 21 kb)

Additional file 4: Data extraction—study characteristics. (DOCX 25 kb)

Additional file 5: Data extraction—study outcomes. (DOCX 19 kb)

Additional file 6: Ongoing studies. (DOCX 16 kb)

Additional file 7: Summary of main findings comparing clowning and midazolam. (PDF 81 kb)

Additional file 8: Summary of main findings comparing clowning and child life. (PDF 70 kb)

## Data Availability

All data generated or analyzed during this study are included in this published article.

## Abbreviations

qRCTs: Quasi-RCTs
RCTs: Randomized controlled trials
RevMan: Review Manager
STAI: State-Trait Anxiety Inventory
