# Supplementary material for: Clowning in children undergoing potentially anxiety-provoking procedures: a systematic review and meta-analysis
Source: Syst Rev. 2019 Jul 19;8:178. doi: 10.1186/s13643-019-1095-4 (PMC6642518; doi:10.1186/s13643-019-1095-4)
Supplement: Supplementary file 2 — Search strategy. (DOCX 20 kb) [file 13643_2019_1095_MOESM2_ESM.docx]

Additional file 2: Search strategy

| MEDLINE via PubMed  (children [tiab] OR child [tiab] OR child [mesh] OR pediatric [tiab] OR paediatric [tiab] OR pediatrics [tiab] OR paediatrics [tiab] OR kids [tiab] OR infant* [tiab] OR infant [mesh] OR adolescent* [tiab] OR adolescent [mesh]) AND clown* [tiab] AND ((Randomized controlled trial [pt] OR controlled clinical trial [pt] OR randomized [tiab] OR randomised [tiab] OR placebo [tiab] OR drug therapy [sh] OR randomly [tiab] OR trial [tiab] OR groups [tiab]) NOT (animals [mh] NOT humans [mh]))   - Performed December 6,2018 with 47 results |
| --- |
| Embase via ELSEVIER  ('child'/exp OR 'child'/de OR 'pediatrics'/exp OR 'pediatrics'/de OR 'paediatrics'/de OR 'infant'/exp OR 'infant'/de OR 'adolescent'/exp OR 'adolescent'/de OR children:ab,ti OR child:ab,ti OR pediatric:ab,ti OR paediatric:ab,ti OR pediatrics:ab,ti OR paediatric:ab,ti OR kids:ab,ti OR infant*:ab,ti OR adolescent*:ab,ti) AND clown*:ab,ti AND (('Randomized Controlled Trial'/exp OR 'Controlled Clinical Trial'/exp OR randomized:ab,ti OR randomised:ab,ti OR placebo:ab,ti OR 'clinical trial'/exp OR randomly:ab,ti OR trial:ab,ti) NOT (animals/exp NOT humans/exp))   - Performed December 6, 2018 with 42 results |
| CENTRAL via Cochrane Library  #1 MeSH descriptor: [Child] explode all trees  #2 MeSH descriptor: [Infant] explode all trees  #3 MeSH descriptor: [Hospitals, Pediatric] explode all trees  #4 MeSH descriptor: [Adolescent] explode all trees  #5 child:ti,ab OR children:ti,ab OR pediatric:ti,ab OR Paediatric:ti,ab OR pediatrics:ti,ab OR paediatrics:ti,ab OR Kids:ti,ab OR Infant*:ti,ab OR adolescent*:ti,ab  #6 #1 OR #2 OR #3 OR #4 OR #5  #7 clown*:ti,ab  #8 #6 AND #7   - Performed December 6, 2018 with 48 results |
