# Supplementary material for: Clowning in children undergoing potentially anxiety-provoking procedures: a systematic review and meta-analysis
Source: Syst Rev. 2019 Jul 19;8:178. doi: 10.1186/s13643-019-1095-4 (PMC6642518; doi:10.1186/s13643-019-1095-4)
Supplement: Supplementary file 3 — Excluded studies. (DOCX 21 kb) [file 13643_2019_1095_MOESM3_ESM.docx]

Additional file 3: Excluded studies

| Study | Reason for exclusion |
| --- | --- |
| Alverson, B. K., Wilson, K. M., & Shah, S. S. (2013). A Randomized Trial of Facilitated Family-Centered Rounds. Hospital pediatrics, 3(2), 156-161. | No potentially fear triggering procedure |
| Antonelli, E., Vagnoli, L., Ciucci, E., Vernucci, C., Lachi, F., & Messeri, A. (2016). A Comparison of Nonpharmacologic Interventions on the Emotional State of Children in the Emergency Department. Pediatric Emergency Care. doi:10.1097/pec.0000000000000900. | Wrong intervention (combined) |
| Ben-Pazi, H., Lotem-Ophir, R., Cohen, A., & Kroyzer-Shapira, N. (2011). Clown care for reducing pain among children with cerebral palsy undergoing needle procedures: The science and art. Developmental Medicine and Child Neurology, 53, 63. | Abstract |
| Ben-Pazi, H., Cohen, A., Kroyzer, N., Lotem-Ophir, R., Shvili, Y., Winter, G., ... & Pollak, Y. (2017). Clown-care reduces pain in children with cerebral palsy undergoing recurrent botulinum toxin injections- A quasi-randomized controlled crossover study. PLoS One, 12(4), e0175028. doi:10.1371/journal.pone.0175028. | Quasi-randomized |
| Ben-Pazi, H., Lotem-Ophir, R., Cohen, A., & Kroyzer-Shapira, N. (2011). Clown care is effective in reducing pain among children with cerebral palsy undergoing botulinum toxin injections. Developmental medicine and child neurology., 2011. 53: p. 20. | abstract |
| Canto, M. A., Quiles, J. M., Vallejo, O. G., Pruneda, R. R., Morote, J. S., Pinera, M. J., . . . Baron, C. (2008). [Evaluation of the effect of hospital clown's performance about anxiety in children subjected to surgical intervention]. Cir Pediatr, 21(4), 195-198. | Not randomized |
| Dionigi, A., Sangiorgi, D., & Flangini, R. (2014). Clown intervention to reduce preoperative anxiety in children and parents: a randomized controlled trial. J Health Psychol, 19(3), 369-380. doi: 10.1177/1359105312471567 | Not randomized |
| Dionigi, A., & Gremigni, P. (2017). A combined intervention of art therapy and clown visits to reduce preoperative anxiety in children. J Clin Nurs, 26(5-6), 632-640. doi:10.1111/jocn.13578. | Wrong intervention (combined) |
| Felluga, M., Rabach, I., Minute, M., Montico, M., Giorgi, R., Lonciari, I., . . . Barbi, E. (2016). A quasi randomized-controlled trial to evaluate the effectiveness of clowntherapy on children’s anxiety and pain levels in emergency department. Eur J Pediatr, *175*(5), 645-650. | Not randomized |
| Fernandes, S. C., & Arriaga, P. (2010). The effects of clown intervention on worries and emotional responses in children undergoing surgery. J Health Psychol, 15(3), 405-415. doi: 10.1177/1359105309350231 | Not randomized |
| Festini, F., Liguori, S., Stacchini, M., Ciofi, D., Giusti, F., Olivini, N., & Bisogni, S. (2014). Effectiveness of a new method to reduce preoperative anxiety in children: Randomised controlled trial. Archives of Disease in Childhood, 99, A79. | Wrong intervention |
| Gorfinkle, K. S., Slater, J. A., Bagiella, E., Tager, F. A., & Labinsky, E. B. (1998). Child Behavioral Distress During Invasive Oncologic Procedures and Cardiac Catheterization With the Big Apple Circus Clown Care Unit• 55. Pediatric Research, 43, 12-12. | No full text available |
| Hansen, L. K., Kibaek, M., Martinussen, T., Kragh, L., & Hejl, M. (2011). Effect of a clown's presence at botulinum toxin injections in children: a randomized, prospective study. J Pain Res, 4, 297-300. doi: 10.2147/jpr.s23199 | Evaluation not based on randomized cases |
| Kocherov, S., Hen, Y., Jaworowski, S., Ostrovsky, I., Gabay, J., Lev, G., . . . Chertin, B. (2015). The influence of the use medical clowns to the reduction of preoperative anxiety, postoperative pain and medical costs in children undergoing outpatient penile surgery: A randomized controlled trial. *Journal of Urology, 193*(4), e173. | abstract only |
| Kristensen, H. N., Lundbye-Christensen, S., Haslund-Thomsen, H., Graven-Nielsen, T., & Elgaard Sorensen, E. (2018). Acute Procedural Pain in Children: Intervention With the Hospital Clown. *Clin J Pain, 34*(11), 1032-1038. | quasi-randomized |
| Liguori, S., Stacchini, M., Ciofi, D., Olivini, N., Bisogni, S., & Festini, F. (2016). Effectiveness of an App for Reducing Preoperative Anxiety in Children: A Randomized Clinical Trial. JAMA pediatrics, 170(8), e160533-e160533. | Wrong intervention |
| Meiri, N., Ankri, A., Hamad-Saied, M., Konopnicki, M., & Pillar, G. (2016). The effect of medical clowning on reducing pain, crying, and anxiety in children aged 2-10 years old undergoing venous blood drawing-a randomized controlled study. Eur J Pediatr, 175(3), 373-379. doi: 10.1007/s00431-015-2652-z | Not randomized |
| Meiri, N., Ankri, A., Ziadan, F., Nahmias, I., Konopnicki, M., Schnapp, Z., . . . Pillar, G. (2017). Assistance of Medical Clowns Improves the Physical Examinations of Children Aged 2-6 Years. *Isr Med Assoc J, 19*(12), 786-791. | quasi-randomized (order of arrival) |
| Meisel, V., Chellew, K., Ponsell, E., Ferreira, A., Bordas, L., & Garcia-Banda, G. (2009). [The effect of "hospital clowns" on distress and maladaptive behaviours of children who are undergoing minor surgery]. Psicothema, 21(4), 604-609. | Not randomized |
| Pinquart, M., Skolaude, D., Zaplinski, K., & Maier, R. F. (2011). Do clown visits improve psychological and sense of physical well-being of hospitalized pediatric patients? A randomized-controlled trial. Klin Padiatr, 223(2), 74-78. doi: 10.1055/s-0030-1267932 | No potentially fear triggering procedure |
| Saliba, F. G., Adiwardana, N. S., Uehara, E. U., Silvestre, R. N., Leite, V. V., Faleiros, F. T., . . . De Gobbi, J. I. (2016). Salivary Cortisol Levels: The Importance of Clown Doctors to Reduce Stress. *Pediatr Rep, 8*(1), 6188. | no potentially fear triggering procedure |
| Scheel, T., Hoeppner, D., Grotevendt, A., & Barthlen, W. (2017). Clowns in Paediatric Surgery: Less Anxiety and More Oxytocin? A Pilot Study. *Klinische Padiatrie, 229*(5), 274-280. | No potentially fear triggering procedure |
| Smerling, A. J., Skolnick, E., Bagiella, E., Rose, C., Labinsky, E., & Tager, F. (1999). Perioperative clown therapy for pediatric patients. Anesthesia & Analgesia, 88(2S), 306S. | No full text available |
| Viggiano, M. P., Giganti, F., Rossi, A., Di Feo, D., Vagnoli, L., Calcagno, G., & Defilippi, C. (2015). Impact of psychological interventions on reducing anxiety, fear and the need for sedation in children undergoing magnetic resonance imaging. Pediatric reports, 7(1), 13-15. | Wrong intervention |
