# Supplementary material for: Clowning in children undergoing potentially anxiety-provoking procedures: a systematic review and meta-analysis
Source: Syst Rev. 2019 Jul 19;8:178. doi: 10.1186/s13643-019-1095-4 (PMC6642518; doi:10.1186/s13643-019-1095-4)
Supplement: Supplementary file 4 — Data extraction—study characteristics. (DOCX 25 kb) [file 13643_2019_1095_MOESM4_ESM.docx]

Additional file 4: Data extraction – study characteristics

| **Study** | **Setting / country / time period** | **Inclusion criteria** | **Procedure** | **Clown intervention** | **Comparison group** | **Children’s age** | **Number of cases (rando-mized)** |
| --- | --- | --- | --- | --- | --- | --- | --- |
| Agostini et al. (2014) | Minor day surgery in hospital / Italy / NR | Age 3–12, Italian nationality, mothers, residents of Cesena, ASA 1-2, absence of prematurity, developmental delay and chronic illness | Anesthesia | Waiting room and during anesthesia induction 🡪 30 minutes  Clown doctor | Parental presence or no intervention: Accompanied by nurse and mother | IG:  5.53 ± 3.34  CG:  4.84 ± 1.95 | 50 |
| Golan et al. (2009) | Elective outpatient surgery / Israel / NR | Age 3-8, ASA 1-2, no previous anesthesia, no chronic illness, no prematurity, no developmental delay, no significant hearing or visual impairments | Anesthesia | Preoperative holding area and throughout induction of anesthesia 🡪 20-30 minutes  Professional clown | CG1:  Parental presence or no intervention  CG2:  No clown but oral midazolam 30 minutes before surgery | Average age 4.5 | 65 |
| Goldberg et al. (2014) | Skin prick test in Hospital / Israel / NR | Age 2-17, no painful medical procedure within 3 months before SPT, no coulrophobia | Allergy skin prick test | Waiting room until skin test was read  Medical clown | Parental presence or no intervention | IG:  8.02 ± 4.65  CG:  8.33 ± 4.58 | 91 |
| Heilbrunn et al. (2014) | University-affiliated children’s hospital / USA/ 11/2011-12/2012 | Age 5-12, nonurgent, no invasive procedure (=any procedure that requires a break in the skin or anatomical orifice) | Physician examination in PED | Occurred before physician entry 🡪 5 to 10 minutes  Hospital clown | CG1:  Child life: 5 to 10 minutes before physician entry  CG2:  Parental presence or no intervention | IG:  7.7 ± 2.3  CG1:  7.4 ± 2.1  CG2:  7.5 ± 2.0 | 120 randomi-zed 113 analyzed |
| Kocherov et al. (2016) | Outpatient surgery / Israel / NR | Age 2-16, ASA level 1-2, scheduled to undergo meatotomy, no previous anaesthetic experience | Anesthesia | Before entering operation room and stay throughout the anesthesia-induction process  Medical clown | Parental presence or no intervention | No difference between ages of children in both groups (p= 0.732) | 80 children enrolled |
| Rimon et al. (2016) | NR / Israel / 9/2014-9/2015 | Age 2-15, mind. one parent present, no urgent IV cannulation, no developmental disabilities, not critically ill, no severe bacterial infection, no glucocorticoids in the last 6 weeks | Venipuncture | Before the procedure  🡪 15 minutes, clown was also present during blood collection  Medical clown | Parental presence or no intervention | IG:  5.6 ± 2.8  CG:  6.9 ± 3.4 | 55 randomi-zed  53 analyzed |
| Vagnoli et al. (2005) | Minor day surgery in hospital / Italy / 6/2003-12/2003 | Age 5-12; ASA level 1-2, residents of Florence, no history of chronic illness, no premature birth, no premedication, no previous anesthesia | Anesthesia | Children accompanied by one parent clowns before entering the operation room and during anesthesia induction 🡪 15 minutes preoperative, later accompanied the child in OR  Clown doctor | Parental presence or no intervention | IG:  7.3 ± 2.72  CG:  6.85 ± 2.21 | 40 |
| Vagnoli et al. (2007) | Minor day surgery in hospital / Italy / 6/2004-3/2005 | Age 5-12, undergoing anesthesia for minor surgery, Italian nationality. ASA 1-2, residents of Florence, no premedication | Anesthesia | Children are accompanied by clown and parent in the operation room and during induction  Not reported | Parental presence or no intervention | IG:  7 ± 2.2  CG:  7.4 ± 2.6 | 50 |
| Vagnoli et al. (2010) | Minor day surgery in hospital / Italy / 4/2009-12/2009 | Age 5-12, ASA 1-2, scheduled to undergo general anesthesia for minor surgery at Anna Meyer Hospital Children’s Hospital in Florence, Italian nationality, residents of Florence and close surrounding, no history of chronic illness, no premature birth, no developmental delay, no previous anesthesia | Anesthesia | Children accompanied by 2 clowns and a parent in the preoperative room, together entering operation room and stay for induction🡪 30 minutes preoperative, during anesthesia 15 minutes  Professional clown | CG2:  Oral midazolam at least 45 minutes before beginning surgical process and presence of 1 parent  CG1:  Parental presence or no intervention | IG:  7 ± 2.2  CG2:  8 ± 2.1  CG1:  7.4 ± 2.6 | 75 randomi-zed |
| Wolyniez et al. (2013) | Emergency department (hospital)/ Israel / 9/2011-9/2012 | Age 3–16, planned blood test or intravenous cannulation, accompanied by at least 1 parent, no urgent intravenous cannulation, no developmental disabilities | Insertion of an intravenous catheter | Before procedure🡪15 minutes  Medical clown | Parental presence or no intervention | IG:  7 ± 4  CG:  8 ± 4 | 47 randomi-zed |
| Yildirim et al. (2018) | Burn unit (hospital) / Turkey / 5/2015 | Age 3-7, first-degree, second-degree or mix type (first and second-degree) burn less than 10% of whole body surface, accompanied by parent during whole procedure of dressing change, no facial and/or genital burn injury, no physical and/or psychological problems, no fear of clowns | Burn dressing change | During burn dressing change  Clown nurse | Parental presence or no intervention | IG: 4.76 +/- 1.36 years  CG: 4.16 +/- 1.1 years | 60 randomi-zed |
