# Supplementary material for: Clowning in children undergoing potentially anxiety-provoking procedures: a systematic review and meta-analysis
Source: Syst Rev. 2019 Jul 19;8:178. doi: 10.1186/s13643-019-1095-4 (PMC6642518; doi:10.1186/s13643-019-1095-4)
Supplement: Supplementary file 5 — Data extraction—study outcomes. (DOCX 19 kb) [file 13643_2019_1095_MOESM5_ESM.docx]

Additional file 5: Data extraction – outcomes

| **Study** | **Anxiety/Pain/Cooperation (children)** | **Measurement (children)** | **Anxiety (parents)** | **Measurement (parents)** |
| --- | --- | --- | --- | --- |
| Agostini et al. (2014) |  |  | STAI Y-1  IG: 35.4 ± 9  CG: 38.4 ± 7.4  STAI Y-2  IG: 34.8 ± 9.7  CG: 38 ± 8.6  VRS-Anxiety  IG: 1.8 ± 1.7  CG: 3.2 ± 1.9 (all p > 0.05) | After separation  STAI 1 + 2 and VRS-Anxiety |
| Golan et al. (2009) | Preoperative:  IG: 28.3 ± 4.6  CG1: 38.4 ± 12.7; IG vs CG1 p=0.0006  CG2: 35.7 ± 11.8; IG vs GC2 p=0.0074  Entrance OR:  IG: 37.3 ± 12.3  CG1: 50.0 ± 17.4; IG vs. CG1 p=0.006  CG2: 42.0 ± 10.6; IG vs GC2 p=0.1802  Mask application:  IG: 62.7 ± 14.6  CG1: 54.4 ± 21.6; IG vs. CG1 p=0.1416  CG2: 49.9 ± 16.0; IG vs GC2 p=0.0062 | m-YPAS | NR | STAI |
| Goldberg et al. (2014) | IG: 31.8 ± 12.1  CG: 45.6 ± 22.8 (p=0.001) | m-YPAS | State anxiety:  IG: 26.9 ± 6.6  CG: 32.3 ± 10 (p=0.004)  Trait anxiety:  IG: 32.0 (no SD reported)  CG: 33.6 (no SD reported) (p=0.4) | STAI 1+2 |
| Heilbrunn et al. (2014) | Patient room T2:  IG: 25.2 ± 3.1  CG1: 23.8 ± 2.2; IG vs. CG1 p=0.0174  CG2: 26.1 ± 4.9; IG vs. CG2 p=0.337  During examination T3:  IG: 25.6 ± 3.4  CG1: 24.4 ± 2.7; IG vs. CG1 p=0.0734  CG2: 25.5 ± 3.4; IG vs. CG2 p=0.9044 | m-YPAS |  |  |
| Kocherov et al. (2016) | Preoperative/postoperative:  No mean or SD reported, just ‘the patients from the first group (clown involvement) demonstrated lower pre-operative anxiety index. P = 0.0319. (Fig. 1) and post-operatively after surgery (P = 0.0042)’ | m-YPAS  (pre- and postoperative) |  |  |
| Rimon et al.. (2016) | 1 minute after the procedure:  IG: 2.2 ± 2.5  CG: 7.5 ± 2.9  (p< 0.001) | FPS-r (4-7 years),  VAS (> 7 years) |  |  |
| Vagnoli et al. (2005) | Waiting room:  IG: 31 ± 11.3  CG: 36 ± 15.6 (p=0.254)  Induction room:  IG: 37.5 ± 21.5  CG: 68.3 ± 28.4 (p=0.000) | m-YPAS | State anxiety  IG: 73.1 ± 25  CG: 77.9 ± 19.2 (p=0.504)  Trait anxiety  IG: 41.5 ± 22.1  CG: 53.3 ± 24.4 (p=0.117) | STAI 1 + 2 |
| Vagnoli et al. (2007) | Waiting room:  IG: 29.8 ± 10.4  CG: 35.6 ± 14.7  (p=0.111)  Induction room:  IG: 34.6 ± 20  CG: 64.5 ± 26.7 (p=0.000) | m-YPAS |  |  |
| Vagnoli et al. (2010) | Waiting room  IG: 29.5 ± 10.5  CG2: 37.4 ± 13.1; IG vs. CG2 p=0.0188  CG1: 35 ±14.4; IG vs. CG1 p=0.1236  Induction room  IG: 33.2 ± 18.8  CG2: 49.7 ± 22.9; IG vs. CG2 p=0.0052  CG1: 65.4 ± 25; IG vs. CG1 p<0.0001 | m-YPAS | State anxiety  IG: 58.5 ± 12.7  CG2: 37.4 ± 13.1; IG vs. CG2 p<0.0001  CG1: 58.3 ± 9.3; IG vs. CG1 p=0.9522  Trait anxiety  IG: 45.5 ± 7.9  CG2: 49.7 ± 22.9; IG vs. CG2 p=0.3788  CG1: 50.3 ± 10.4; IG vs. CG1 p=0.0644 | STAI 1+2 immediately after separation and during operation |
| Wolyniez et al. (2013) |  |  | IG: 31 ± 12  CG: 37 ± 13 (p=0.14) | STAI after procedure |
| Yildirim et al. (2018) | Total score (0-16; 16 worst compliance)  IG: 4.8 ± 4.2  CG: 11 ± 4.6 p<0.001 | Questionnaire & Child Observation Form during procedure |  |  |
