# Supplementary material for: Clowning in children undergoing potentially anxiety-provoking procedures: a systematic review and meta-analysis
Source: Syst Rev. 2019 Jul 19;8:178. doi: 10.1186/s13643-019-1095-4 (PMC6642518; doi:10.1186/s13643-019-1095-4)
Supplement: Supplementary file 6 — Ongoing studies. (DOCX 16 kb) [file 13643_2019_1095_MOESM6_ESM.docx]

Additional file 6: Ongoing studies

| **Study ID** | **Outcomes** | **Comparison group** | **Potentially fear triggering procedure** | **Author request** |
| --- | --- | --- | --- | --- |
| NCT02199587 | Children’s and parental anxiety, changes in hormones | Parental presence | Endocrine test | No answer |
| NCT00886314 | Preoperative children’s and parental anxiety | Midazolam and parental presence | Minor surgery undergoing general anesthesia | No Answer |
| NCT01622218 | Children’s pain, use of analgesics, cytokine levels | Parental presence | Hernia repair surgery | Clown seems to have no impact on children |
| NCT02701322 | Patient’s collaboration, subjective experience, quality of examination | No intervention | Videofluoroscopic examination of pediatric speech disorder | Study ceased due to technical issues |
| NCT02668679 | Children’s anxiety | Parental presence | Digestive endoscopic procedure | No answer |
| NCT03122015 | Acceptability, children’s pain, children’s anxiety | No intervention | Painful procedure at infectious diseases clinic | No sufficient contact information available |
| NCT03324828 | Children’s anxiety, cortisol level (blood), cortisol level (salive), | Placebo + clowns, Placebo + no clowns, Hydroxzine + clowns, Hydroxzine + no clowns | Anesthesia | No answer |
| NCT03671317 | Children’s anxiety, children’s pain | No intervention | Venipuncture | No request as study was not yet recruiting |
