# Supplementary material for: Clowning in children undergoing potentially anxiety-provoking procedures: a systematic review and meta-analysis
Source: Syst Rev. 2019 Jul 19;8:178. doi: 10.1186/s13643-019-1095-4 (PMC6642518; doi:10.1186/s13643-019-1095-4)
Supplement: Supplementary file 7 — Summary of main findings comparing clowning and midazolam. (PDF 81 kb) [file 13643_2019_1095_MOESM7_ESM.pdf]

Summary of findings:

Clowning compared to Midazolam in children undergoing potentially anxiety-provoking procedures

Patient or population: children undergoing potentially anxiety-provoking procedures

Setting: any setting

Intervention: clowning

Comparison: Midazolam

Outcomes	Anticipated absolute effects* (95% CI)		Relative effect (95% CI)	№ of participants (studies)	Certainty of the evidence (GRADE)	Comments
	Risk with Midazolam	Risk with clowning				
Children's anxiety during preoperative time assessed with: m-YPAS Scale from: 0 to 100		The mean children's anxiety during preoperative time in the intervention group was 7.6 lower (11.73 lower to 3.47 lower)	-	93 (2 RCTs)	⊕◯◯◯ VERY LOW [a,b,c,d,e,f]	
Children's anxiety in induction room assessed with: m-YPAS Scale from: 0 to 100		The mean children's anxiety in induction room in the intervention group was 9.63 lower (21.04 lower to 1.77 higher)	-	93 (2 RCTs)	⊕◯◯◯ VERY LOW [a,b,c,d,e,f,g]	
Children's anxiety during mask application assessed with: m-YPAS Scale from: 0 to 100		The mean children's anxiety during mask application in the intervention group was 12.8 higher (3.65 higher to 21.95 higher)	-	43 (1 RCT)	⊕◯◯◯ VERY LOW [a,b,d,e,f,h]	
Parental anxiety - State anxiety assessed with: STAI Y-1 Scale from: 20 to 80		The mean parental anxiety - State anxiety in the intervention group was 21.1 higher (13.95 higher to 28.25 higher)	-	50 (1 RCT)	⊕◯◯◯ VERY LOW [a,b,c,d,f,i]	
Parental anxiety - Trait anxiety assessed with: STAI Y-2 Scale from: 20 to 80		The mean parental anxiety - Trait anxiety in the intervention group was 4.2 lower (13.7 lower to 5.3 higher)	-	50 (1 RCT)	⊕◯◯◯ VERY LOW [a,b,c,d,f,i]	

***The risk in the intervention group** (and its 95% confidence interval) is based on the assumed risk in the comparison group and the **relative effect** of the intervention (and its 95% CI).

CI: Confidence interval; MD: Mean difference

Summary of findings:

Clowning compared to Midazolam in children undergoing potentially anxiety-provoking procedures

Patient or population: children undergoing potentially anxiety-provoking procedures

Setting: any setting

Intervention: clowning

Comparison: Midazolam

Outcomes	Anticipated absolute effects* (95% CI)		Relative effect (95% CI)	№ of participants (studies)	Certainty of the evidence (GRADE)	Comments
	Risk with Midazolam	Risk with clowning				

GRADE Working Group grades of evidence
High certainty: We are very confident that the true effect lies close to that of the estimate of the effect
Moderate certainty: We are moderately confident in the effect estimate: The true effect is likely to be close to the estimate of the effect, but there is a possibility that it is substantially different
Low certainty: Our confidence in the effect estimate is limited: The true effect may be substantially different from the estimate of the effect
Very low certainty: We have very little confidence in the effect estimate: The true effect is likely to be substantially different from the estimate of effect

Explanations

a. Unclear risk of selection bias across the studies reporting this outcome.

b. High risk of performance bias across the studies reporting this outcome.

c. High risk of detection bias across the studies reporting this outcome.

d. Unclear risk of attrition bias across the studies reporting this outcome.

e. High risk of reporting bias across the studies reporting this outcome.

f. Sample size less than 400

g. Substantial heterogeneity

h. Unclear risk of detection bias across the studies reporting this outcome.

i. Unclear risk of reporting bias across the studies reporting this outcome.
