# Supplementary material for: Clowning in children undergoing potentially anxiety-provoking procedures: a systematic review and meta-analysis
Source: Syst Rev. 2019 Jul 19;8:178. doi: 10.1186/s13643-019-1095-4 (PMC6642518; doi:10.1186/s13643-019-1095-4)
Supplement: Supplementary file 8 — Summary of main findings comparing clowning and child life. (PDF 70 kb) [file 13643_2019_1095_MOESM8_ESM.pdf]

Summary of findings:

Clowning compared to child life program in children undergoing potentially anxiety-provoking procedures

Patient or population: children undergoing potentially anxiety-provoking procedures

Setting: any setting

Intervention: clowning

Comparison: child life program

Outcomes	Anticipated absolute effects* (95% CI)		Relative effect (95% CI)	№ of participants (studies)	Certainty of the evidence (GRADE)	Comments
	Risk with child life program	Risk with clowning				
Children's anxiety in patient room assessed with: m-YPAS Scale from: 0 to 100		The mean children's anxiety in patient room in the intervention group was 1.4 higher (0.25 higher to 2.55 higher)	-	84 (1 RCT)	⨁◯◯◯ VERY LOW [a,b,c,d]	
Children's anxiety during physician examination assessed with: m-YPAS Scale from: 0 to 100		The mean children's anxiety during physician examination in the intervention group was 1.2 higher (0.11 lower to 2.51 higher)	-	84 (1 RCT)	⨁◯◯◯ VERY LOW [a,b,c,d]	

***The risk in the intervention group** (and its 95% confidence interval) is based on the assumed risk in the comparison group and the **relative effect** of the intervention (and its 95% CI).

CI: Confidence interval; **MD:** Mean difference

GRADE Working Group grades of evidence
High certainty: We are very confident that the true effect lies close to that of the estimate of the effect
Moderate certainty: We are moderately confident in the effect estimate: The true effect is likely to be close to the estimate of the effect, but there is a possibility that it is substantially different
Low certainty: Our confidence in the effect estimate is limited: The true effect may be substantially different from the estimate of the effect
Very low certainty: We have very little confidence in the effect estimate: The true effect is likely to be substantially different from the estimate of effect

Explanations

a. High risk of performance bias across the studies reporting this outcome.

b. High risk of attrition bias across the studies reporting this outcome.

c. Unclear risk of reporting bias across the studies reporting this outcome.

d. Sample size less than 400
